# Objective Assessment of Counselling for Fetal Heart Defects: An Interdisciplinary Multicenter Study

**DOI:** 10.3390/jcm9020467

**Published:** 2020-02-08

**Authors:** Alexander Kovacevic, Stefan Bär, Sebastian Starystach, Andreas Simmelbauer, Michael Elsässer, Andreas Müller, Aida Mohammadi Motlagh, Renate Oberhoffer-Fritz, Eva Ostermayer, Peter Ewert, Matthias Gorenflo, Annette Wacker-Gussmann

**Affiliations:** 1 Department of Pediatric and Congenital Cardiology, Heidelberg University Hospital, 69120 Heidelberg, Germany; simmelbauer@dhm.mhn.de (A.S.); A.Mueller@med.uni-heidelberg.de (A.M.); Matthias.Gorenflo@med.uni-heidelberg.de (M.G.); 2 Max Weber Institute for Sociology, Ruprecht Karls University Heidelberg, 69115 Heidelberg, Germany; stefan.baer@mwi.uni-heidelberg.de (S.B.); sebastian.starystach@soziologie.uni-heidelberg.de (S.S.); 3 Department of Gynecology and Obstetrics, Heidelberg University Hospital, 69120 Heidelberg, Germany; Michael.Elsaesser@med.uni-heidelberg.de; 4 Institute of Preventive Pediatrics, Faculty of Sport and Health Sciences, Technical University of Munich, 80992 Munich, Germany; aida_motlagh@yahoo.de (A.M.M.); oberhoffer@dhm.mhn.de (R.O.-F.); annette.wacker-gussmann@tum.de (A.W.-G.); 5 German Heart Center Munich, Department of Pediatric Cardiology and Congenital Heart Defects, 80636 Munich, Germany; ewert@dhm.mhn.de; 6 Department of Obstetrics and Gynecology, Klinikum rechts der Isar, Technical University of Munich, 81675 Munich, Germany; eva.ostermayer@t-online.de

**Keywords:** fetal heart disease, antenatal diagnosis, counselling, interdisciplinary study, Pediatric Cardiology, Fetal Cardiology, Maternal-Fetal Medicine, Perinatology, sociology

## Abstract

The objective of this study was to analyze parental counselling for fetal heart disease in an interdisciplinary and multicenter setting using a validated questionnaire covering medical, sociodemographic, and psychological aspects. *n* = 168 individuals were recruited from two pediatric heart centers and two obstetrics units. Overall, counselling was combined successful and satisfying in >99%; only 0.7% of parents were dissatisfied. “Perceived situational control” was impaired in 22.6%. Adequate duration of counselling leads to more overall counselling success (*r* = 0.368 ***), as well as providing written or online information (57.7% vs. 41.5%), which is also correlated to more “Transfer of Medical Knowledge” (*r* = 0.261 ***). Interruptions of consultation are negatively correlated to overall counselling success (*r* = −0.247 **) and to “Transparency regarding the Treatment Process” (*r* = −0.227 **). Lacking a separate counselling room is associated with lower counselling success for “Transfer of Medical Knowledge” (*r* = 0.210 ***). High-risk congenital heart disease (CHD) is correlated to lower counselling success (42.7% vs. 71.4% in low-risk CHD). A lack of parental language skills leads to less overall counselling success. There is a trend towards more counselling success for “Transfer of Medical Knowledge” after being counselled solely by cardiologists in one center (*r* = 0.208). Our results indicate that a structured approach may lead to more counselling success in selected dimensions. For complex cardiac malformations, counselling by cardiologists is essential. Parental “Perceived Situational Control” is often impaired, highlighting the need for further support throughout the pregnancy.

## 1. Introduction

The number of high-risk pregnancies has dramatically increased over recent decades, with congenital heart disease (CHD) being the most common cause of congenital malformations. The birth prevalence is reported to be around 9 per 1000 live births [1]. Due to technical developments and improved education of sonographers, fetal/pediatric cardiologists, and maternal fetal medicine (MFM) specialists, the prenatal detection rate of cardiac abnormalities has improved continuously. The postnatal survival rate has increased since then, in particular in lesions that are duct dependent [2,3,4,5].

The task of fetal/pediatric cardiologists or maternal fetal medicine (MFM) specialists is not only to make an accurate diagnosis of the malformation and its effects on hemodynamics, but also to counsel parents appropriately. An explanation of available treatment options and prognostic aspects will help parents to make decisions which are best for them. This includes the discussion of termination of pregnancy (TOP) or perinatal palliative care, if indicated [6].

Although this topic has a great impact in the field of fetal cardiology, relatively little effort has been made concerning research focused specifically on providing the most effective counselling techniques [7]. Existing recommendations for fetal cardiologists, including parental counselling, do not seem to be fully sufficient. Counselling practices even seem to fail to cover certain topics [7,8,9].

Therefore, the objective of our prospective and interdisciplinary multicenter approach was to objectively assess parental counselling for fetal heart disease, explore parental needs and potentially affecting factors for successful counselling, and suggest modifications ultimately aiming at improvements for this service. The aim was not only to cover topics on information transfer on CHD, treatment, and prognosis, but also to consider sociodemographic and psychological aspects.

## 2. Materials and Methods

To assess parental counselling comprehensively, we used a pretested and validated questionnaire, developed after interdisciplinary collaboration between cardiologists, MFM specialists, and sociologists [10].

For the questionnaire, Likert scaled and open questions were combined with socio-psychological aspects and sociodemographic data.

An objective assessment of “Counselling success” (= dependent variable) was performed by creating five analytical dimensions as described by our group earlier [10]. Pretesting showed sufficient internal consistency; further methodical aspects are published elsewhere [10].

These dimensions are:

1. “Transfer of Medical Knowledge”;

2. “Trust in Medical Staff”;

3. “Transparency Regarding the Treatment Process”;

4. “Coping Resources”;

5. “Perceived Situational Control”.

The dependent variable overall “Counselling success” was constructed by building a sum score of the 16 items from the Likert scale in the questionnaire and transforming it into an ordinal scaled variable defining the respective range 16–32 as successful, 33–63 as satisfying, and 64–80 as unsuccessful. The items themselves were constructed as indicators showing success when answered positively in the two assenting values of the Likert response format 1–5. In the same way, we constructed the variables for the five dimensions of counselling success, whereby the number of items varied (except the dimension “Perceived Situational Control” that was measured by only one item).

Questions on informational, temporal, spatial, or social topics served as independent variables.

CHD was graded according to severity and correlations to counselling success were calculated [6].

We identified cases with a prenatally diagnosed CHD after database analyses in two large pediatric heart centers in Germany. Fetal echocardiography and counselling were performed in two heart centers or two obstetric units by three fetal/pediatric cardiologists and by three MFM specialists. Counselling was performed uniformly by experienced consultant cardiologists and/or MFM specialists, either separately or together.

The data were collected from November 2016–August 2018 (center one) and from November 2018–October 2019 (center two) by issuing questionnaires during outpatient appointments or by interviews. Parents were interviewed by two coauthors (A.S. and A.M.M.) who were not involved in diagnosing prenatal CHD or counselling parents to avoid response bias.

Accordingly, this is a quantitative study with retro- and prospective data acquisition.

Statistical analysis was performed using IBM^®^ SPSS^®^ Statistics Version 25 (calculations of average, standard deviation, and median, as well as correlation, cross tabulation, and frequency analyses).

All subjects gave their written informed consent for inclusion before they participated in the study. The study was conducted in accordance with the Declaration of Helsinki, and the protocol was approved by the local Ethics Committee of each involved Medical Faculty (Center one: date 17.06.2016; reference number: S-250/2016; center two: date 10.09.2018; reference number: 341/18 S-KK).

## 3. Results

### 3.1. Reliability Analyses

Internal consistency of the questionnaire was tested showing sufficient values for Cronbach’s alpha for the five created analytical dimensions (Cronbach’s alpha = reliability coefficient with values from 0 to 1.0; >0.7 shows sufficient and >0.8 good internal consistency of the created scale; Table 1).

### 3.2. Descriptive Analyses

From two heart centers and two obstetric units, *n* = 168 parents were recruited (*n* = 100 female, median age 34 years, range 19–46 years/*n* = 68 male, median age 35 years, range 26–52 years). Median gestational age at first counselling was 23 weeks (range 12–38 weeks).

Counselling was performed by cardiologists in 61.1%, and MFM specialists in 38.9%. Sixty percent of parents felt that the time was short between the suspected cardiac diagnosis and consultation by a specialist. The majority of parents were worried (64.2%) and uncertain about the situation (51.4%). Also, 73.8% of them considered the situation of fetal CHD to be serious.

Furthermore, 80.1% felt that the duration of the consultation was appropriate, and 33.3% received written information after counselling. However, 11.5% stated that there were frequent interruptions during counselling.

### 3.3. Overall Counselling Success as per Definition and in the Five Analytical Dimensions

Overall, counselling was successful in 47.3% and satisfying in 52.1%; 0.7% of parents were dissatisfied (Table 2).

Looking at counselling success in the different dimensions, “Perceived Situational Control” was considered unsuccessful in 22.6% of cases, whereas for the remainder, counselling was combined successful and satisfying in at least 95% or higher (Table 2).

### 3.4. Effect of Duration and Interruptions of Counselling

Adequate length (neither too short nor too long) of consultation was positively correlated to overall counselling success (*r* = 0.368 ***), and to all five analytical dimensions (Transfer of Medical Knowledge: *r* = 0.398 ***; Trust in Medical Staff: 0.282 ***; Transparency regarding the Treatment Process: *r* = 0.405 ***; Coping resources: *r* = 0.247 **; Perceived Situational Control: *r* = 0.299 ***).

**Figure 1:**
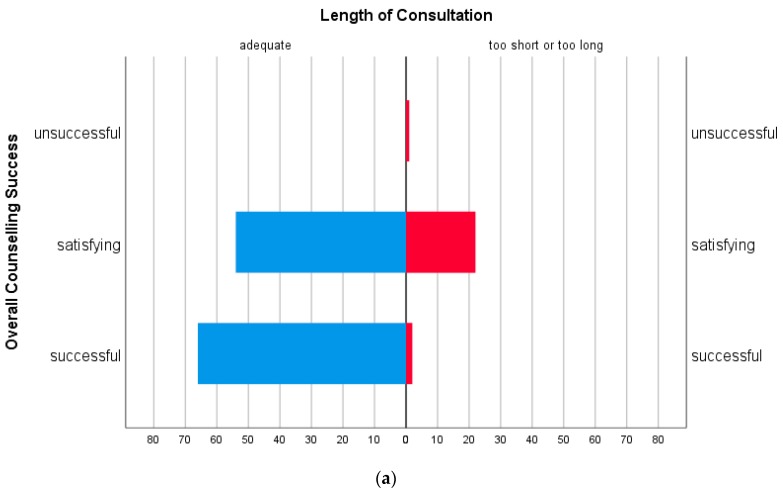

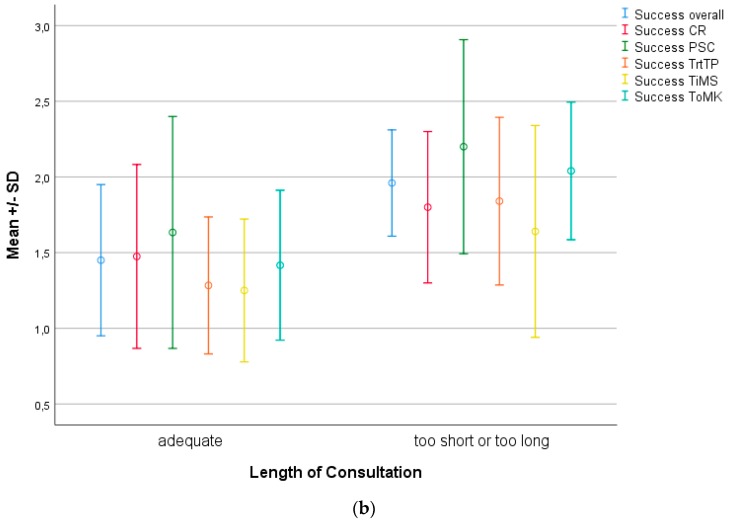
(**a**) Overall Counselling Success differentiated according to whether the length of the consultation was perceived as appropriate or not. The columns show the number of nominations. (**b**) Overall Counselling Success and Counselling Success in the five dimensions surveyed (mean value and standard deviation-SD) differentiated according to whether the length of the consultation (without time spent for fetal echocardiography) was perceived as appropriate or not (1 = successful to 3 = unsuccessful). Abbreviations: CR—Coping Resources, PSC—Perceived Situational Control, TrtTP—Transparency Regarding the Treatment Process, TiMS—Trust in Medical Staff, ToMK—Transfer of Medical Knowledge.

Figure 1a illustrates the high rate for overall counselling success if the length of the consultation was perceived as appropriate.

Figure 1b shows, in addition, more counselling success in the five dimensions if the length of the consultation was perceived as appropriate.

Interruptions during consultations were negatively correlated to overall counselling success (*r* = −0.247 **) and to “Transparency regarding the Treatment Process” (*r* = −0.227 **).

### 3.5. Informational Aspects

Providing additional written information or links to online sources explaining the nature, therapy, and outcomes of the heart defect led to more overall counselling success and more counselling success in all five analytical dimensions (Table 3). Furthermore, it was significantly correlated to more counselling success for “Transfer of Medical Knowledge” (*r* = 0.261 ***) and “Perceived Situational Control” (*r* = 0.229 **).

### 3.6. Influence of a Separate Counselling Room

Lacking a separate room for counselling was significantly correlated to lower counselling success in the dimension “Transfer of Medical Knowledge” (*r* = −0.210 **).

### 3.7. Counselling Success Depending on Severity of CHD

In cases with high-risk CHD (58% of all CHD in this study; see supplementary Table S1 for details on CHD, grading, and associated genetic or extracardiac anomalies), overall counselling success was lower, i.e., 42.7%, compared to low risk CHD (71.4%). Analyses of the five dimensions show that when comparing low- and high-risk CHD, counselling success was always lower in the high-risk group, with the most significant differences for “Coping Resources” and “Transparency regarding the Treatment Process”. However, “Perceived Situational Control” was better in the high-risk compared to the low-risk group (Table 4).

### 3.8. Influence of German as Native Language

A decrease of counselling success overall and in all five analytical dimensions was observed if the parental native language was not German, with the most significant differences for “Transfer of Medical Knowledge”, “Trust in Medical Staff ”, and “Transparency regarding the Treatment Process” (Table 5; cases were only included if parental German language skills were adequate during conversation; no cases were included if counselling was performed via a translator as an objective assessment of translation quality would not have been feasible).

### 3.9. Counselling Success Depending on Who Has Counselled and How Often

No deviation from a random distribution of counselling success was found by cross table analyses if cardiologists did not counsel parents. However, there is a trend towards more counselling success for “Transfer of Medical Knowledge” after being counselled solely by cardiologists in one center (*r* = 0.208).

The absolute number of counselling sessions did not affect counselling success significantly overall or in any analytical dimension.

### 3.10. Counselling Success Depending on the Institution

No deviation from a random distribution of “Counselling success” was found (overall or in any dimension) when comparing the two University hospitals.

## 4. Discussion

Higher detection rates of critical fetal cardiac malformations and incremental parental demand have made counselling of parents after diagnosis of fetal CHD increasingly challenging. In contrast, little effort has been made in training physicians for parental counselling, and counselling is not standardized among tertiary centers. Further, research in this field is patchy, as there is no standardized approach to analyze objectively counselling for fetal heart disease.

In this multicenter study, we demonstrate specific parental needs and affecting factors for counselling success after prenatal diagnosis of CHD, which may provide guidance to those who work in such programs as to what they can do to improve their service.

A pretested, standardized questionnaire was used to assess the dependent variable “Effective Counselling”. We were able to combine data from two large pediatric heart centers and obstetric units in Germany, which helped to increase statistical power. The interdisciplinary approach with cardiologists, MFM specialists, and sociologists involved is an additional specific strength of our work, and together with the multicenter aspect, this effort appears to be exceptional in the existing literature. It has been shown earlier that multidisciplinary studies are potentially very useful, leading to improvements in treatment quality, patient compliance, and outcomes [11].

In our study, overall counselling was combined successful and satisfying in >99%. “Perceived situational control” was often impaired. This indicates the parental need for a continued support by e.g., cardiac nurse specialists, psychologists or social workers. However, a current general lack of nursing staff in Germany poses a potential problem in the realization of this demand [12].

Adequate length of consultations was positively correlated to overall counselling success and to all five analytical dimensions, whereas interruptions were negatively correlated to overall counselling success and “Transparency regarding the Treatment Process”.

The variable “length of the consultation” was measured in Likert format with five characteristics ranging from “too short” to “too long”. In this respect, it is the subjective perception of the length of the consultation, not an objective time measurement. The mean value of “neither too short nor too long” was used as an indicator for an appropriate length. This indicator does not necessarily have to correlate with the satisfaction with the counselling, because this was measured over five dimensions with a multiplicity of items as counselling success, and it was therefore the empirical question for us whether and to what extent counselling success was connected with the perception that the length of the consultation was reported to be too short, too long, or appropriate.

It is likely that the adequate duration of counselling has to be estimated by the counsellor and parents to find the individual optimum. This may change after repeated counselling sessions.

The lack of a separate counselling room led to less counselling success for “Transfer of Medical Knowledge”. This should be considered when setting up a Fetal Cardiology service to plan a room specifically intended for counselling.

Additional written information was valued as well as providing adequate online information sources, both leading to more overall counselling success. Furthermore, we found a positive correlation to “Transfer of Medical Knowledge” and “Perceived situational control”. This highlights the need to have adequate written information and/or online links prepared for parents, at least for the second appointment.

We could demonstrate that the severity of fetal CHD may be an issue for counselling success. If high-risk CHD was present, overall counselling success was lower (42.7%) compared to low-risk CHD (71.4%). In particular counselling success for “Coping resources” and “Transparency regarding the Treatment Process” was lower in the high-risk CHD group, indicating a need for improvement. Early parental support by e.g., psychologists may increase success for “Coping resources” in high-risk CHD.

Repetitive counselling did not affect overall counselling success or any dimension positively, in contrast to previously published data [13,14]. However, we could demonstrate a trend for more counselling success for “Transfer of Medical Knowledge” after being counseled by cardiologists in one center. In view of the finding that high-risk CHD seems to be problematic, we believe that this again indicates the necessity for parental counselling by cardiologists in particular for complex CHD. The AEPC has stated in their guidelines that counselling for CHD must be provided by cardiologists, but can be done in conjunction with obstetricians or neonatologists [8]. In our experience, both strategies seem feasible in achieving counselling success, in particular combined counselling by cardiologists, MFM specialists, and neonatologists, e.g., in the presence of extracardiac anomalies, but always by a cardiologist if CHD is diagnosed.

In our study, parental language difficulties, i.e., a mother tongue other than German, which were unapparent during routine conversation, led to less overall counselling success and in all five dimensions, which is in line with previously published data [14]. Timely inclusion of translators may lead to more counselling success in selected cases.

### 4.1. Literature Review

Recommendations of parental counselling for fetal heart defects have been published by different associations and societies with varying key aspects [7,8,15,16]. The topics that need to be covered are well described there. In addition, the option of a fetal cardiac intervention in selected cases should be discussed [17,18,19].

Generally, nondirective counselling is recommended to avoid parents being influenced by a professional’s personal attitude [20,21]. The medical information provided should help parents to make their own decisions. These include the options to continue the pregnancy, to terminate the pregnancy, or to decide for perinatal palliative care. For the latter, a national structured perinatal palliative care program would be the optimum setup. However, such programs are missing in many European countries [22]. Regarding TOP, it has been shown that it is more often chosen in situations involving high-risk CHD and complex extracardiac malformations [23]. Effective counselling is likewise essential in cases of low-risk CHD and intended TOP by parents.

Specific analyses of parental counselling after fetal diagnosis of CHD show evidence that published recommendations do not lead to counselling success with certainty. Cardiologists often provide different information than that required by parents. In particular, the child’s quality of life after potential multiple cardiac surgeries seems underrepresented during counselling [9]. In complex CHD, such as fetal Hypoplastic Left Heart Syndrome (HLHS), long-term complications including neurodevelopmental issues are often infrequently addressed [24].

Further, parents appreciate a short waiting time for an appointment with a specialist, and afterwards, repetitive counselling sessions. Written information and illustrations of CHD after initial counselling are valued [13,14]. The benefits of web sites as an information source is controversially discussed. Carlsson et al. found that the information provided online is often of low quality and unspecific, or the opposite, i.e., too complex for parents, whereas high-quality web-sites may be appreciated [13,14].

Our results, together with the literature review, indicate that there seems to be a demand to optimize counselling practices. There is further potential benefit if structured teaching for cardiologists and MFM specialists can be implemented, e.g., based on our assessment of counselling. Finally, our methodological approach might help to establish further research tools for parental counselling for CHD, not only after prenatal diagnosis.

### 4.2. Summary

From this data and the literature, we can conclude that for an optimized program, counselling should be performed shortly after the fetal echocardiogram in a separate room.

Consultations should not be disturbed or interrupted by any other personnel.

It may be useful to start with an open question by asking parents what they understand so far of the cardiac diagnosis, especially if the fetus has earlier been seen in another center.

After an initial structured explanation of the CHD, adequate demonstration material (such as printed diagrams) seems to be helpful to improve parental understanding (including the explanation of a “normal” heart).

During the conversation, parental language competence must be acknowledged; this includes also a subtle language barrier.

Consultants should undergo specific training to improve their communication skills. They should also be able to effectively convey multiple complex diagnoses (e.g., a combination of CHD with extracardiac anomalies and genetic syndromes). The ability to assess parental understanding should be considered important for training purposes, because their abilities to absorb information may be impaired in this emotional situation. A respectful interaction with the parents’ feelings is mandatory. This includes recognizing a parental “right not to know”.

In general, the consultation may be divided into two parts with a short break in between. First, key aspects of the cardiac diagnoses should be provided: the anatomy of the heart defect, potential limitations of the prenatal diagnosis, and aspects of postnatal therapy (and prenatal in selected cases), including a discussion of potential complications after surgery and known data on long-term sequelae. High-quality illustrative material is shown to be very useful to explain heart defects and therapeutic options.

After a short break which gives parents the possibility to process the information, emerging questions should be discussed, followed by specific questions, if parents feel there is a need for further support (e.g., by psychologists, social-workers). Parents should be encouraged to express their sorrows, fears, and uncertainties.

Finally, hand-outs with information on the diagnosis (including information on appropriate websites) and contact data are important, as is arranging further follow-up appointments to see other specialists such as geneticists, cardiac surgeons, or others.

### 4.3. Limitations

Different socio-economic and educational realms may have a significant influence on counselling success. Furthermore, religious, cultural, and ethnic backgrounds may influence parental decision-making. Specific data is not included in this manuscript. This is an interesting topic that will be considered in the forthcoming analysis.

It can be assumed that skills regarding counselling differ among professionals. This variable was not analyzed in the present study, because one aim was to determine differences in counselling success depending on the medical specialty, not between professionals in general.

Further, it cannot be ruled out that personal bias was imposed during counselling, although in all centers included for this study, nondirective counselling is the standard approach.

## 5. Conclusions

Parental counselling for fetal heart disease is a complex, multidimensional process.

Our results indicate that a structured approach may lead to improved counselling success in selected dimensions, such as “Transfer of Medical Knowledge” or “Transparency regarding the Treatment Process”, if the duration of the counselling is adequate, if it takes place in a separate room without interruptions, and if inadequate parental language skills, even if unapparent, are acknowledged. As complex CHD seems to be particularly problematic, it appears to be consequent that parents need to be counselled by cardiologists for cardiac malformations. Parental “Perceived Situational Control” is often impaired, highlighting the need for further support throughout the pregnancy.

Analyses of parental sociodemographic aspects seem worthwhile in order to identify modifiers of counselling success, and thus make further improvements for this service. Initial analyses on this question show that neither age, gender, educational level, nor social status have a substantial influence on the counselling situation. In contrast, social aspects turn out to be crucial, such as experiencing support during the counselling and perceiving that certain questions and concerns are being adequately addressed.

## Figures and Tables

**Table 1:** Internal consistency of the questionnaire (Cronbach’s alpha = reliability coefficient with values from 0 to 1.0; >0.7 shows sufficient and >0.8 good internal consistency of the created scale).

Counselling Success	
overall	0.905
Transfer of Medical Knowledge	0.792
Trust in Medical Staff	0.792
Transparency regarding Treatment Process	0.809
Coping resources	0.770
Sorrows	0.815

**Table 2:** Overall counselling success and in the five created analytical dimensions.

	Counselling Success in %		
	Successful	Satisfying	Unsuccessful
Overall	47.3	52.1	0.7
Transfer of Medical Knowledge	47.6	50.6	1.8
Trust in Medical Staff	70.6	26.3	3.1
Transparency regarding the Treatment Process	63.4	35.4	1.2
Coping resources	50.3	44.7	5.0
Perceived Situational Control	43.9	33.5	22.6

**Table 3:** Providing additional written information or links to online information sources leads to more overall counselling success and counselling success in all five created analytical dimensions.

	Written or Online Information:	
	Provided	Not Provided
Overall Counselling Success	57.7%	41.5%
Transfer of Medical Knowledge	65.5%	38.5%
Trust in Medical Staff	78.2%	66.7%
Transparency regarding Treatment Process	76.8%	59.3%
Coping resources	59.3%	45.8%
Perceived situational control	61.8%	34.9%

**Table 4:** Overall counselling success and in all five analytical dimensions depending on the severity of congenital heart disease (CHD).

	Degree of CHD Severity		
	1	2	3
overall	71.4 %	47.5 %	42.7 %
Transfer of Medical Knowledge	61.1 %	43.5 %	45.4 %
Trust in Medical Staff	88.2 %	66.7 %	69.5 %
Transparency regarding Treatment Process	88.9 %	55.3 %	62.5 %
Coping resources	76.5 %	47.7 %	46.4 %
Perceived situational control	36.8 %	37.8 %	47.4 %

**Table 5:** Overall counselling success and in all five analytical dimensions depending on the parental native language.

	Native Language	
	German	Not German
overall	48.8 %	40.9 %
Transfer of Medical Knowledge	50.0 %	37.0 %
Trust in Medical Staff	74.6 %	48.1 %
Transparency regarding Treatment Process	64.7 %	52.0 %
Coping resources	50.7 %	50.0 %
Perceived situational control	44.4 %	38.5 %

## Supplementary Materials

The following are available online at https://www.mdpi.com/2077-0383/9/2/467/s1, Table S1: Details on CHD, grading, and associated genetic or extracardiac anomalies.

## References

[1] Van der Linde D., Konings E.E., Slager M.A., Witsenburg M., Helbing W.A., Takkenberg J.J., Roos-Hesselink J.W. (2011). Birth prevalence of congenital heart disease worldwide: A systematic review and meta-analysis. J. Am. Coll. Cardiol..

[2] Bonnet D., Coltri A., Butera G., Fermont L., Le Bidois J., Kachaner J., Sidi D. (1999). Detection of transposition of the great arteries in fetuses reduces neonatal morbidity and mortality. Circulation.

[3] Franklin O., Burch M., Manning N., Sleeman K., Gould S., Archer N. (2002). Prenatal diagnosis of coarctation of the aorta improves survival and reduces morbidity. Heart.

[4] Tworetzky W., McElhinney D.B., Reddy V.M., Brook M.M., Hanley F.L., Silverman N.H. (2001). Improved surgical outcome after fetal diagnosis of hypoplastic left heart syndrome. Circulation.

[5] Holland B.J., Myers J.A., Woods C.R. (2015). Prenatal diagnosis of critical congenital heart disease reduces risk of death from cardiovascular compromise prior to planned neonatal cardiac surgery: A meta-analysis. Ultrasound Obstet. Gynecol..

[6] Allan L.D., Huggon I.C. (2004). Counselling following a diagnosis of congenital heart disease. Prenat. Diagn..

[7] Donofrio M.T., Moon-Grady A.J., Hornberger L.K., Copel J.A., Sklansky M.S., Abuhamad A., Cuneo B.F., Huhta J.C., Jonas R.A., Krishnan A. (2014). Diagnosis and treatment of fetal cardiac disease: A scientific statement from the American Heart Association. Circulation.

[8] Allan L., Dangel J., Fesslova V., Marek J., Mellander M., Oberhänsli I., Oberhoffer R., Sharland G., Simpson J., Sonesson S.E. (2004). Fetal Cardiology Working Group; Recommendations for the practice of fetal cardiology in Europe. Association for European Paediatric Cardiology. Cardiol. Young.

[9] Arya B., Glickstein J.S., Levasseur S.M., Williams I.A. (2013). Parents of children with congenital heart disease prefer more information than cardiologists provide. Congenit. Heart Dis..

[10] Kovacevic A., Simmelbauer A., Starystach S., Elsässer M., Sohn C., Müller A., Bär S., Gorenflo M. (2018). Assessment of Needs for Counseling After Prenatal Diagnosis of Congenital Heart Disease—A Multidisciplinary Approach. Klin. Padiatr..

[11] Bertsche T., Neininger M.P., Kaune A., Schumacher P.M., Dumeier H.K., Bernhard M.K., Syrbe S., Kiess W., Merkenschlager A., Bertsche A. (2018). Interdisciplinary Concepts of Paediatrics and Clinical Pharmacy to Optimise Anticonvulsant Treatment. Klin. Padiatr..

[12] Maibach-Nagel E. (2018). Pflegekräftemangel: Ein wirklich großes Thema. Dtsch. Arztebl..

[13] Carlsson T., Bergman G., Wadensten B., Mattsson E. (2016). Experiences of informational needs and received information following a prenatal diagnosis of congenital heart defect. Prenat. Diagn..

[14] Bratt E.L., Järvholm S., Ekman-Joelsson B.M., Mattson L.Å., Mellander M. (2015). Parent’s experiences of counselling and their need for support following a prenatal diagnosis of congenital heart disease—A qualitative study in a Swedish context. BMC Pregnancy Childbirth..

[15] Paladini D., Alfirevic Z., Carvalho J.S., Khalil A., Malinger G., Martinez J.M., Rychik J., Gardiner H. (2016). Prenatal counseling for neurodevelopmental delay in congenital heart disease: Results of a worldwide survey of experts’ attitudes advise caution. Ultrasound Obstet. Gynecol..

[16] Paladini D., Alfirevic Z., Carvalho J.S., Khalil A., Malinger G., Martinez J.M., Rychik J., Ville Y., Gardiner H., ISUOG Clinical Standards Committee (2017). ISUOG consensus statement on current understanding of the association of neurodevelopmental delay and congenital heart disease: Impact on prenatal counseling. Ultrasound Obstet. Gynecol..

[17] Kovacevic A., Öhman A., Tulzer G., Herberg U., Dangel J., Carvalho J.S., Fesslova V., Jicinska H., Sarkola T., Pedroza C. (2018). Fetal hemodynamic response to aortic valvuloplasty and postnatal outcome: A European multicenter study. Ultrasound Obstet. Gynecol..

[18] Friedman K.G., Sleeper L.A., Freud L.R., Marshall A.C., Godfrey M.E., Drogosz M., Lafranchi T., Benson C.B., Wilkins-Haug L.E., Tworetzky W. (2018). Improved technical success, postnatal outcome and refined predictors of outcome for fetal aortic valvuloplasty. Ultrasound Obstet. Gynecol..

[19] Tulzer A., Arzt W., Gitter R., Prandstetter C., Grohmann E., Mair R., Tulzer G. (2018). Immediate effects and outcome of in-utero pulmonary valvuloplasty in fetuses with pulmonary atresia with intact ventricular septum or critical pulmonary stenosis. Ultrasound Obstet. Gynecol..

[20] Malhotra A., Menahem S., Gillam L. (2010). Ethical issues in fetal management: A cardiac perspective. Int. J. Pediatr..

[21] Menahem S., Grimwade J. (2004). Counselling strategies in the prenatal diagnosis of major heart abnormality. Heart Lung Circ..

[22] Flaig F., Lotz J.D., Knochel K., Borasio G.D., Führer M., Hein K. (2019). Perinatal Palliative Care: A qualitative study evaluating the perspectives of pregnancy counselors. Palliat. Med..

[23] Nell S., Wijngaarde C.A., Pistorius L.R., Slieker M., ter Heide H., Manten G.T., Freund M.W. (2013). Fetal heart disease: Severity, associated anomalies and parental decision. Fetal Diagn. Ther..

[24] Walsh M.J., Verghese G.R., Ferguson M.E., Fino N.F., Goldberg D.J., Owens S.T., Pinto N., Zyblewski S.C., Quartermain M.D. (2017). Counseling Practices for Fetal Hypoplastic Left Heart Syndrome. Pediatr. Cardiol..

